# Synergistic Effects of Capric Acid and Colistin against Colistin-Susceptible and Colistin-Resistant *Enterobacterales*

**DOI:** 10.3390/antibiotics12010036

**Published:** 2022-12-26

**Authors:** Yi-Yun Liu, Zong-Hua Qin, Hui-Ying Yue, Phillip J. Bergen, Li-Min Deng, Wan-Yun He, Zhen-Ling Zeng, Xian-Feng Peng, Jian-Hua Liu

**Affiliations:** 1 National Risk Assessment Laboratory for Antimicrobial Resistance of Animal Original Bacteria, Guangdong Provincial Key Laboratory of Veterinary Pharmaceutics Development and Safety Evaluation, South China Agricultural University, Guangzhou 510642, China; 2 Guangdong Laboratory for Lingnan Modern Agriculture, Guangzhou 510642, China; 3 Guangzhou Insighter Biotechnology Co., Ltd., Guangzhou 510642, China; 4 Biomedicine Discovery Institute, Department of Microbiology, School of Biomedical Sciences, Monash University, Clayton, VIC 3800, Australia

**Keywords:** colistin resistance, combination therapy, capric acid, synergy

## Abstract

Colistin is a last-line antibiotic against Gram-negative pathogens. However, the emergence of colistin resistance has substantially reduced the clinical effectiveness of colistin. In this study, synergy between colistin and capric acid was examined against twenty-one Gram-negative bacterial isolates (four colistin-susceptible and seventeen colistin-resistant). Checkerboard assays showed a synergistic effect against all colistin-resistant strains [(FICI, fractional inhibitory concentration index) = 0.02–0.38] and two colistin-susceptible strains. Time–kill assays confirmed the combination was synergistic. We suggest that the combination of colistin and capric acid is a promising therapeutic strategy against Gram-negative colistin-resistant strains.

Global antimicrobial resistance poses a serious threat to human health. Over the last decade, there has been a rapid increase in the number of multidrug-resistant (MDR) and extensively drug-resistant (XDR) Gram-negative bacteria [1,2]. Of particular concern is the emergence of carbapenem-resistant Gram-negative pathogenic bacteria such as *Acinetobacter baumannii*, *Escherichia coli*, *Pseudomonas aeruginosa* and *Klebsiella pneumoniae* which represent a special clinical challenge [3,4]. This situation has only been made worse by the slow development of new antibiotics and the rapid spread of drug-resistant genes, which have led to a decline in the number of effective antibiotics available to treat infections caused by MDR bacteria. Thus, the emergence of such pathogens including carbapenemase-producing *Enterobacterales* has resulted in the reintroduction of colistin as a last-resort antibiotic treatment [5]. Colistin, a polymyxin antibiotic discovered in the 1950s, is a cationic polypeptide antibiotic produced by *Bacillus polymyxa* which acts as a bactericidal agent by targeting the polyanionic lipid A of lipopolysaccharide (LPS) in the outer membrane of Gram-negative bacteria [6]. However, widespread use of this agent has given rise to a remarkable increase in colistin-resistant strains [7]. Polymyxin resistance was previously thought to be due solely to mutations in chromosomal genes which led to the modification of lipid A with phosphoethanolamine (pEtN) and/or 4-amino-4-deoxy-L-arabinose (L-Ara4N), thereby reducing the interaction of lipid A with polymyxins [7]. This paradigm changed when we reported for the first time plasmid-mediated colistin resistance via the *mcr-1* gene in an isolate from China [8]. Subsequently, more than 60 countries and regions have demonstrated the presence of *mcr-1*-harboring strains [9], with other *mcr* family genes (*mcr-2* to *mcr-10*) having since been reported worldwide [10]. The *mcr* genes encode a pEtN transferase that modifies lipid A with pEtN residues [10]. These reports have led to global concern regarding the efficacy of colistin, raising an urgent need for the identification of new antimicrobial compounds to address these challenges.

Synergy with colistin has been investigated for a variety of traditional antimicrobial agents including rifampicin, rifabutin, carbapenems, clarithromycin, novobiocin, macrolides, minocycline, tigecycline, and glycopeptides [11]. MacNair, C. R et al., demonstrated that when used in combination with colistin, the best synergistic killing activity was achieved with rifampicin, novobiocin, rifabutin, minocycline, and clarithromycin [11]. Synergy with polymyxins has also been shown with several FDA-approved non-antibiotic drugs, representing another promising alternative treatment pathway that is currently underexplored [12,13,14]. Of these, particularly noteworthy are recent studies that have described synergy against a variety of MDR Gram-negative bacteria with colistin in combination with the signal peptidase inhibitor MD3, the antiretroviral HIV drugs zidovudine and azidothymidine, as well as curcumin, netropsin, and auranofin [15,16,17,18]. For example, Martinez-Guitian et al., confirmed that not only did the combination of MD3 and colistin increase the susceptibility of colistin-susceptible clinical isolates of *A. baumannii*, but that potent synergy with this combination was also observed against colistin-resistant strains with mutations in *pmrB* and phosphoethanolamine modification of lipid A [15]. Similarly, Feng et al., reported potent synergy when colistin was combined with auranofin against problematic colistin-resistant Gram-negative bacteria both in vitro (*K. pneumoniae*, *A. baumannii*, *E. coli* and *Pseudomonas aeruginosa*) and in vivo (*K. pneumoniae*, *A. baumannii*) [18]. Altogether, these results suggest that the use of colistin in combination with such compounds has the potential to enhance bacterial killing of MDR pathogens and prolong the utility of this increasingly important last-line antibiotic. Accordingly, the aim of this study was to find an agent with potential synergistic activity with colistin and assess the in vitro activity of the combination against colistin-susceptible and -resistant Gram-negative bacterial isolates. 

We initially screened over 200 compounds for antimicrobial activity against a colistin-resistant *E. coli* strain, with capric acid identified as a candidate compound. Capric acid is a saturated free fatty acid (FFA) with 10 carbon atoms in the carbon chain and a carboxyl group (–COOH) at one end and a methyl group (–CH_3_) at the other [19]. FFAs are found in marine organisms and plants and have been reported to show antimicrobial activity by targeting the cell membrane and causing damage to cellular energy production and enzyme activity [20,21]. Although capric acid has been shown to have killing activity against the Gram-positive organism *Cutibacterium acnes* [22] and the fungus *Candida albicans* cells [23], activity against Gram-negative organisms has not been investigated. We examined the synergistic potential of capric acid in combination with colistin in vitro against 21 Gram-negative bacterial isolates. 

Four colistin-susceptible and seventeen colistin-resistant strains were tested (Table 1), comprising *K. pneumoniae* (n = 7), *E. coli* (n = 7), *Salmonella* (n = 5), and *Pseudomonas aeruginosa* (n = 2). Of the colistin-resistant strains, twelve carried the *mcr-1* gene, three the *mcr-8* gene, while two harbored mutations in chromosomal genes associated with colistin-resistance. The minimum inhibitory concentrations (MICs) of colistin and capric acid determined for all strains using broth dilution as per the Clinical Laboratory Standards Institute (CLSI) guidelines (M07-A11) are shown in Table 1. The colistin MICs of the colistin-resistant strains were ≥4 mg/L, while the colistin MICs of susceptible strains ranged from 0.5 to 1 mg/L. The MICs of capric acid were all ≥3200 mg/L, suggesting capric acid had no antimicrobial activity against the tested strains when used as monotherapy. 

Synergy between colistin and capric acid was then evaluated by checkerboard assay in a 96-well microtiter plate as previously described [24]. In brief, serial 2-fold dilutions of colistin and capric acid were undertaken with the final concentrations ranging from 0.06 to 32 mg/L for colistin and from 25 to 3200 mg/L for capric acid. Each test organism was added to a density of 5 × 10^6^ CFU/mL, with the final volume in each well being 200 μL. The 96-well plate was then incubated at 37 °C for 18 h. The fractional inhibitory concentration index (FICI) for the interaction of the combination was calculated as follows: FICI = FIC of drug A + FIC of drug B = [(MIC of drug A in combination/MIC of drug A alone) + (MIC of drug B in combination/MIC of drug B alone)]. The FICI was interpreted as follows: FICI ≤ 0.5, synergy; FICI > 0.5–4, indifference; FICI > 4, antagonism. The antibacterial activity of colistin increased when used in combination with capric acid, with synergy observed against 19 of the 21 strains, including against all four bacterial species examined (FICI values ranged from 0.02 to 0.38; Table 1). Importantly, the combination of colistin and capric acid was synergistic against all colistin-resistant strains. The only two strains for which synergy was not observed were *Salmonella* SH47 (FICI, 0.51) and *P. aeruginosa* 10104 (FICI, 2) (Table 1). 

To further examine the potential for synergy with capric acid combined with an antibiotic, we repeated the checkerboard study above using two *E. coli* isolates (2D-8 and SHP50) and ciprofloxacin, cefotaxime, neomycin, tetracycline, and ampicillin. As shown in Table 2, synergy between capric acid and other antimicrobials was not observed (FICI range, 1–2).

Based on the checkerboard study results, the potential for enhanced bacterial killing with the combination of colistin (0.5× MIC) and capric acid (800 mg/L) was examined using time-kill studies against a colistin-susceptible reference strain (*E. coil* C600) and *mcr-1*-positive colistin-resistant strain (*E. coli* C600 + pHNSHP45) according to a previously reported method [25]. The starting inoculum was ~10^6^ CFU/mL and experiments were conducted for 36 h. Antibiotic-free Mueller-Hinton broth served as the control. Synergy was considered to be a ≥2-log_10_ reduction in CFU/mL with the combination when compared to the most active monotherapy at the specified time [26]. Bacterial cultures (5 mL) were incubated with shaking (180 rpm) at 37 °C and samples (5 mL) collected at 0, 2, 4, 8, 24, 28, 32 and 36 h for viable counting. The results of time–kill studies are shown in Figure 1. For both strains, no bacterial killing was observed with capric acid monotherapy and growth mirrored that of the growth control. For the colistin-susceptible reference strain (*E. coli* C600), initial bacterial killing of ~4 log_10_ CFU/mL with colistin monotherapy at 4 h was followed by rapid regrowth that had returned to control values by 28 h (Figure 1A). Amplification of colistin-resistant subpopulations in heteroresistant isolates, namely susceptible isolates based upon their MICs but which contain resistant subpopulations, is known to contribute to regrowth following polymyxin monotherapy [27]. However, synergy was observed with combination therapy from 4 h onwards such that no viable bacteria were observed across 4–8 h, with subsequent regrowth remaining at ~5 log_10_ CFU/mL below that of the control and monotherapies. Synergistic killing was also observed from 8 h onwards with the *mcr-1*-positive colistin-resistant strain (*E. coli* C600 + pHNSHP45), although the enhancement of bacterial killing with the combination was less than that observed with *E. coli* C600 (Figure 1B). Altogether, the time-kill experiments showed good bactericidal activity (≥3 log_10_ CFU/mL) and synergy with combination therapy against both strains (Figure 1). 

Resistance genes are often associated with various mobile genetic structures, facilitating their dissemination among different *Enterobacterales* species. The plasmid-mediated transmission of the colistin resistance gene *mcr-1* has raised serious concerns for the efficacy of colistin, pointing to the urgent need for new agents to effectively treat clinical infections caused by colistin-resistant isolates [8]. Here, we demonstrated synergistic bacterial killing with the combination of colistin and capric acid against a variety of *mcr-1*-positive Gram-negative bacterial strains (Table 1 and Figure 1). While the specific mechanism(s) for the observed synergy with this combination is not yet known, one possible explanation is that capric acid disturbs the stability and activity of the bacterial membrane, enabling more colistin to target lipid A. Like other FFAs, capric acid can cross the cell membrane and disrupt the electron transport chain, resulting in a reduction of ATP production [19]. It has previously been shown that capric acid has anti-adhesion activity and inhibits the adhesion of *C. albicans* cells to abiotic surfaces [23]. A recent study by Jakub et al., showed that capric acid combined with either fluconazole or amphotericin B achieved synergistic killing of *C. albicans* by causing the MDR transporter Cdr1p to relocalize from the plasma membrane to the interior of the cell, leading to reduced efflux activity of Cdr1p [28]. These findings raise the possibility that capric acid may have an impact on the function/integrity of bacterial membranes, thereby enabling more colistin to bind lipid A and thus increasing bacterial susceptibility to colistin. 

Another possible mechanism for the synergy between colistin and capric acid involves the prevention of lipid A modifications by capric acid. The bactericidal activity of colistin begins when it binds to the lipid A component of LPS. The LPS structure can be altered via two-component regulatory systems (TCSs, e.g., PmrAB, ParRS, CprRS, PhoPQ and CrrAB) [7]. The TCSs are sensitive to environmental stimuli including exposure to exogenous compounds or metal ions such as Mg^2+^ and Fe^2+^, which usually results in the modification of the lipid A phosphate groups via the addition of cationic L-Ara4N and/or pEtN moieties [7]. MCR-1 is phosphoethanolamine (pEtN) transferase that leads to the addition of pEtN to lipid A. It is possible that exogenous capric acid prevents such modifications of lipid A, thereby retaining sensitivity to colistin. However, further studies specifically examining the mechanism(s) of synergy are required.

In conclusion, we have demonstrated that capric acid can enhance bacterial killing of colistin-resistant Gram-negative bacteria when combined with colistin. Although the underlying mechanism(s) of synergy with this combination remains to be elucidated, our results suggest that further exploration of this combination against MDR pathogens is warranted. 

## Figures and Tables

**Figure 1:**
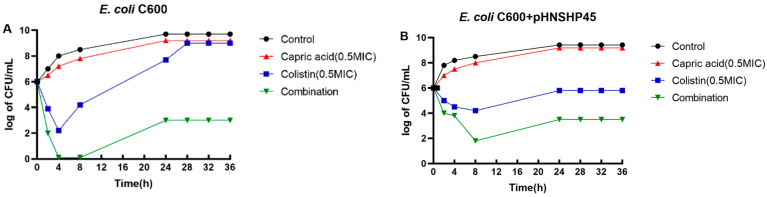
Time-kill study performed with *E. coli* C600 (**A**) and *E. coli* C600 + pHNSHP45 (**B**) with colistin monotherapy (0.5× MIC), capric acid monotherapy (800 mg/L), and their combination (equivalent concentrations).

**Table 1:** FICI and MIC (mg/L) values for the strains tested in this study.

Strain	Origin	PCR for *mcr-1* ^a^	MIC (mg/L)				FICI ^b^
			Single Drug		Combination		
			Colistin	Capric Acid	Colistin	Capric Acid	
*K. pneumoniae* P11	Human	-	1	>3200	0.13	400	0.26
*K. pneumoniae* P11+ pHNSHP45	Transformant	+	8	>3200	0.5	200	0.13
*K. pneumoniae* 117	Pig	+	16	>3200	2	400	0.26
*K. pneumoniae* 281	Pig	+	16	>3200	1	200	0.13
*K. pneumoniae* SDQ8C53R ^c^	Chicken	-	>512	3200	1	50	0.02
*K. pneumoniae* HNJ9C285 ^c^	Chicken	-	>512	3200	4	200	0.07
*K. pneumoniae* HNJ9C245 ^c^	Chicken	-	>512	3200	2	100	0.04
*E. coli* C600	-	-	0.5	>3200	0.125	200	0.26
*E. coli* C600 + pHNSHP45	Transformant	+	8	>3200	0.5	200	0.13
*E. coli* 2D-8 ^d^	Pig	-	8	>3200	0.5	200	0.13
*E. coli* SHP7	Pig	+	8	>3200	2	200	0.31
*E. coli* SHP8	Pig	+	8	>3200	1	200	0.19
*E. coli* SHP50	Pig	+	8	>3200	0.5	200	0.13
*E. coli* GDF36	Fish	+	8	3200	1	800	0.38
*Salmonella* SA316	Pig	+	8	>3200	1	400	0.26
*Salmonella* SH271	Pig	+	4	3200	1	50	0.27
*Salmonella* SH138	Pig	+	8	>3200	1	400	0.26
*Salmonella* SH47	Pig	-	1	>3200	0.25	800	0.51
*Salmonella* SH17	Pig	+	4	3200	1	50	0.27
*P. aeruginosa* 10104	CVCC ^f^	-	1	>3200	1	>3200	2.00
*P. aeruginosa* 10104 (R) ^e^	Induced	-	8	>3200	1	400	0.26

^a^ + and - indicate positive and negative for *mcr-1*. ^b^ FICI—Fractional Inhibitory Concentration Index. ^c^ *K. pneumoniae* strains harboring colistin resistance gene *mcr-8*. ^d^ *E. coli* 2D-8 harbors mutations in *phoP* (Y114I), *phoQ* (E232D), and *pmrA* (L3S). ^e^ *P. aeruginosa* 10104 (R) harbors mutations in *phoQ* (K151R), *parS* (T222R), *colR* (L1M), and *cprR* (T171P). ^f^ CVCC—China Veterinary Culture Collection Center.

**Table 2:** MIC (mg/L) values of capric acid and various antibiotics and FICI values of capric acid when combined with each antibiotic against two colistin-resistant *E. coli* strains.

Strain		Antibiotic					
		Colistin	Ciprofloxacin	Cefotaxime	Neomycin	Tetracycline	Ampicillin
*E. coli* 2D-8	MIC ^a^	8	0.5	0.06	256	32	128
	FICI ^b^	0.13	2	2	1	1	1
*E. coli* SHP50	MIC ^a^	8	32	0.5	0.25	128	8
	FICI ^b^	0.19	1	2	1	1	1

^a^ MIC of the single drug. ^b^ FICI—Fractional Inhibitory Concentration Index.

## Data Availability

Not applicable.
